# Didymin may Enhance Tendon‐Bone Healing Partly by Promoting PPAR‐Mediated Fatty Acid Oxidation and Macrophage M2 Polarization

**DOI:** 10.1002/iid3.70485

**Published:** 2026-09-29

**Authors:** Xiaojun Ma, Jia Shen, Jun Ma, Hangyu Li, Yang Li, Jun Wan

**Affiliations:** ^1^ Sports Medicine Department, People's Hospital of Ningxia Hui Autonomous Region Third Clinical Medical College of Ningxia Medical University Yinchuan China; ^2^ Intensive Care Unit General Hospital of Ningxia Medical University Yinchuan China

**Keywords:** didymin, fatty acid oxidation pathway, macrophage, tendon bone healing

## Abstract

**Objective:**

Achieving rapid and high‐quality regeneration after injury at the tendon‐bone interface remains an urgent challenge in the fields of orthopedics, sports medicine, and tissue regeneration. Didymin (DID) possesses a range of pharmacological activities, including anti‐inflammatory, antioxidant, and antimicrobial properties; however, there is a research gap regarding the role of DID in tendon‐bone healing. Therefore, this study intends to further investigate the role of DID in tendon‐bone healing.

**Methods:**

A tendon‐bone healing mouse model of Achilles tendon—Achilles bone was constructed, and the mice were treated with different doses of DID. The therapeutic effect of DID on tendon‐bone healing and its effect on macrophage polarization were investigated by evaluating various parameters such as tendon‐bone healing, fibrocartilage formation at the tendon‐bone interface, and macrophage polarization. In addition, in vitro experiments were used to verify whether the PPAR‐mediated fatty acid oxidation (FAO) pathway was involved in the induction of macrophage polarization by DID.

**Results:**

Treatment with DID was found to promote fibrocartilage formation in tendon‐bone junction tissues, ameliorate pathological injuries, induced macrophage polarization towards the M2 type, and downregulate the levels of IL‐1β and IL‐6, while upregulating the levels of Arg1, IL‐10, CoA, PPAR, and RXRA. However, the addition of a PPAR‐γ antagonist (GW9662) or an FAO inhibitor (Etomoxir) significantly attenuated the effects of DID on macrophage polarization.

**Conclusion:**

This study demonstrates that DID may enhance histological and molecular features of tendon‐bone healing partly through PPAR‐mediated fatty acid oxidation and macrophage M2 polarization, thereby facilitating tendon bone healing, with potential application value.

## 1 Introduction

Tendon‐bone insertion injury refers to injuries to the attachment sites of tendons and bone tissues, including rotator cuff injuries and Achilles tendon injuries, which are frequently encountered in daily life and sports [1, 2, 3]. Studies have reported that the incidence of tendon and bone injuries is increasing annually, and it is challenging to restore normal physiological function [4]. Tendon‐bone healing is a process of tissue healing and reconstruction between soft and hard tissues. Due to its complex anatomical structure and its inability to regenerate tissues after injury, tendon‐bone healing has become a key issue in sports medicine and orthopedic research [5, 6]. Postoperative re‐rupture rates remain high in clinical tendon‐bone injuries, resulting in increased treatment burdens [7]. Consequently, achieving rapid and high‐quality regeneration following tendon‐bone damage remains an urgent challenge in orthopedics, sports medicine, and tissue regeneration.

Research confirms that the regeneration of the fibrocartilage layer following injury at the bone‐tendon interface is closely related to the quality of tissue repair. Promoting fibrocartilage formation during tendon‐bone healing is key to improving healing quality [8]. Macrophages play a crucial role throughout the entire repair process at the bone‐tendon interface. In the early stage of healing, there is a large number of local M1 macrophage infiltrations, which secrete a large number of pro‐inflammatory factors to enhance the local inflammatory response, and stimulate cell division and chemotaxis of fibroblasts to gather at the damaged site. Subsequently, macrophages polarized to the M2 type, secreted a large number of anti‐inflammatory factors to reduce the local inflammatory response and promote tissue regeneration and repair [9, 10]. It has been shown that the transformation of the tendon‐bone interface microenvironment from a pro‐inflammatory to an anti‐inflammatory state is crucial for the smooth transition from the inflammatory to the proliferative phase, and that macrophage polarization phenomena play a pivotal role in the transformation of the local inflammatory microenvironment [11]. It has been demonstrated that using biological interventions to induce early aggregation of M2‐type macrophages at the tendon‐bone interface promotes tendon‐bone healing [12]. In addition, studies have reported that M2 macrophages are dependent on oxidative phosphorylation (OXPHOS) and fatty acid oxidation (FAO) for energy, and that activation of the peroxisome proliferator‐activated receptor (PPAR) and its regulatory genes contributes to an increase in FAO [13, 14]. Therefore, inducing metabolic reprogramming of macrophages toward FAO and OXPHOS to achieve M2 polarization is significant for tendon bone healing.

Didymin (DID) is the most abundant dihydroflavonoid constituent of *Clinopodium chinense* (Benth.) [15]. Reports have shown that DID has purgative, anti‐inflammatory, and antioxidant properties [16]. Moreover, DID modulates the M1/M2 macrophage balance by converting pro‐inflammatory M1‐like macrophage phenotypes to anti‐inflammatory M2‐like phenotypes without altering M2‐like macrophage polarization [17]. Metabolic tracing revealed that DID enhances FAO rather than glycolysis pathways [18]. Although DID has a wide range of functions, its role in tendon‐to‐bone healing remains largely unexplored. The ability of DID to drive the transformation of macrophages from the M1 to the M2 phenotype suggests its potential in promoting tendon‐to‐bone healing.

In this study, we established a mouse model of tendon‐bone healing and gavage of mice with different concentrations of DID. Using this model, we investigated the effect of DID on interfacial bone formation and structure, fibrocartilage formation, and macrophage polarization modulation. Furthermore, cellular experiments verified the role of the PPAR signaling‐mediated FAO pathway. The results are expected to reveal important avenues for treating and preventing tendon‐bone healing.

## 2 Materials and Methods

### 2.1 Materials

Didymin (DID, Cat No. TC1639‐231128) was purchased from Sichuan RIRENI Technology Co. Ltd. (China). EDTA (Cat No. YE0105, Bomei, Hefei, China). Disulfiram (DSF, Cat No. HY‐B0240), PPAR‐γ antagonist (GW9662, Cat No. HY‐16578), and fatty acid oxidation inhibitor (Etomoxir, Cat No. HY‐50202) were purchased from MedChem Express (China). Hematoxylin and eosin (H&E, Cat No. H9627) was purchased from Sigma‐Aldrich (USA). Safranin O‐fast green (Cat No. G1371) was bought from Solarbio (Beijing, China). Erythrocyte lysate (Cat No. G2015‐500ML) was bought from Servicebio (Wuhan, China). The kits used to quantify calcium (Ca, Cat No. C004‐2‐1) level were purchased from Nanjing Jiancheng Biotechnology Company (China). The kits used to quantify Osteocalin (OCN, Cat No. ZC‐38207), alkaline phosphatase (ALP, Cat No. ZC‐33964), and Acetyl‐CoA (Cat No. ZC‐38946) levels were purchased from Shanghai ZCI Bio (China). The APC anti‐mouse F4/80 (Cat No. 123115), PE anti‐mouse CD86 (Cat No. 159203), and FITC anti‐mouse CD206 (MMR, Cat No. 141703) were purchased from Biolegend (China). The Sox9 (Cat No., BS‐10725R), Collagen II (Cat No., bsm‐33409M), F4/80 (Cat No. 263101), CD86 (Cat No. bs‐1035R), and CD206 (Cat No. bsm‐60761R) antibodies were bought from Bioss (China). TB Green Premix Ex Taq Ⅱ (Tli RNaseH Plus) (Cat No. RR820A) provided by Takara (China). Western blot analysis of PPAR‐γ (Cat No. A11183) and RXRA (Cat No. A19105) primary antibodies was purchased from ABclonal (Wuhan, China).

### 2.2 Animals and Experimental Protocol

A total of 36 SPF‐grade male C57BL/6 J mice (body weight 18 ~ 22 g) were obtained from the Chengdu Dossy Experimental Animals Co. Ltd. (License No.: SCXK (Chuan) 2019‐028). All experimental protocols were reviewed and approved by the animal ethics committee of the People's Hospital of Ningxia Hui Autonomous Region, with approval number [2023]‐NZR‐134. After the mice were acclimatized and fed for 1 week, they were randomly assigned to six groups:

- sham‐operated group (sham, saline, *n* = 6)
- model group (saline, *n* = 6)
- Didymin low dose group (l‐DID, 1 mg/kg/d, *n* = 6)
- Didymin medium dose group (M‐DID, 2 mg/kg/d, *n* = 6)
- Didymin high dose group (H‐DID, 4 mg/kg/d, *n* = 6)
- positive drug group (DSF, 50 mg/kg/d, *n* = 6)

A tendon‐bone healing model for the Achilles tendon‐Achilles bone in mice was established by referring to the methods as described in the literature [19]. Briefly, mice were anaesthetized by intraperitoneal injection of 1% sodium pentobarbital, and the hind limbs and feet were disinfected on both sides with 75% alcohol. The skin was incised longitudinally centered on the Achilles tendon, the tissue was dissected to expose the Achilles tendon and the Achilles bone, the Achilles tendon was severed with a No. 11 blade close to the upper edge of the Achilles bone, and the blade scraped off the residual fibrocartilaginous layer on the Achilles tendon. At the center of the posterior border of the Achilles, a bone channel was drilled from back to front with a BD Insulin Syringe (29 G) needle, and a 6‐0 suture with thread was passed through the channel, and the tip of the needle passing through the broken end of the Achilles tendon to be sutured, and then the broken end of the Achilles tendon was sutured and secured to the upper border of the Achilles bone by tying a knot on the suture at the other end of the bone channel. After the Achilles tendon was firmly repaired, the surgical incision was closed and sterilized. The wound was disinfected with iodophor daily for 3 days after the operation, and local penicillin was injected to prevent infection. In the sham‐operated group, only the Achilles tendon and Achilles bone were exposed without being cut. After 1 day of surgery, the mice were gavaged daily for 4 weeks with the corresponding doses of DID and DSF [18, 20], while the sham and model groups received equal volumes of saline. After the last administration, blood was collected from the abdominal aorta of mice. For the sacrifice, mice were anesthetized with an intraperitoneal injection of pentobarbital sodium (50 mg/kg, Sinopharm Chemical Reagent Co. Ltd., Shanghai, China) and sacrificed by decapitation. A portion of the tendon‐bone junction tissue was fixed in 4% buffered formaldehyde solution, while the other part was stored at −80°C for subsequent analysis.

### 2.3 Cell Experiment

The mouse tendon‐bone junction tissues were rinsed with PBS, bone marrow was collected, and a bone marrow cell suspension was prepared; the bone marrow cell suspension was filtered through a 200‐mesh sieve to obtain single‐cell suspension; the single‐cell suspension was centrifuged at 1000 rpm for 5 min, and the supernatant was discarded. The cells were resuspended with erythrocyte lysate at 4°C and centrifuged at 1000 rpm for 5 min, and the bone marrow cells were then obtained. Complete medium containing macrophage colony stimulating factor (M‐CSF) was prepared (DMEM (containing 4.5 g sugar, l‐glutamine, 20 mM HEPES) + 15% FBS + 20 ng/mL M‐CSF + 50 μM 2‐mercaptoethanol). The above extracted bone marrow cells were resuspended in 10 mL of complete medium containing M‐CSF, and the cell concentration was adjusted to 2 × 10^6^ cells/mL; 5 mL of cell suspension was added to a 60 mm cell culture dish, and the cells were cultured in an incubator at 37°C with 5% CO_2_ to obtain mature mouse bone marrow–derived macrophages (BMDMs) for the subsequent experiments.

Setting cell groupings:

i.
  - BMDMs (M0)
  - BMDMs + LPS/IFN‐γ (M1)
  - BMDMs + LPS/IFN‐γ + DID (30 μM)

ii.
  - BMDMs (M0)
  - BMDMs + IL‐4/IL‐13 (M2)
  - BMDMs + IL‐4/IL‐13 + DID (30 μM)

iii.
  - BMDMs (M0)
  - BMDMs + LPS/IFN‐γ (M1)
  - BMDMs + LPS/IFN‐γ + DID (30 μM)
  - BMDMs + LPS/IFN‐γ + DID (30 μM) + PPAR‐γ antagonist (GW9662, 5 μM)
  - BMDMs + LPS/IFN‐γ + DID (30 μM) + fatty acid oxidation inhibitor (Etomoxir, 50 μM)

BMDMs were treated with LPS (100 ng/mL) + IFN‐γ (20 ng/mL) or IL‐4 (20 ng/mL) + IL‐13 (20 ng/mL), respectively, for 24 h. Then, the corresponding DID, GW9662, and Etomoxir were added and treated for 24 h.

### 2.4 Micro CT Scanning

The sample was scanned using a Skyscan 1176 Micro CT with the following parameters: voxel size: 17.76 μm, camera pixel size: 12.59 μm, source voltage: 65 kV, source current: 385 μA, and a 1 mm aluminum alloy plate was used as the filter. The obtained data were reconstructed and analyzed using CTAn, CTvol, CTvox software, and the data were collected. The microstructural parameters of each sample were counted and calculated: percent bone volume (BV/TV), bone surface (BS), trabecular thickness (Tb. Th), trabecular number (Tb. N), trabecular separation (Tb. Sp), and bone mineral density (BMD).

### 2.5 Hematoxylin–Eosin (HE) and Safranin‐O/Fast Green Staining

The tissues were fixed in 4% paraformaldehyde for 24 h. The tissues were transferred into the configured 10% EDTA decalcification solution (Bomei, Hefei, China), and the decalcification effect was observed every 3 days and replaced with fresh decalcification solution until the decalcification was completed. Then the tissues were dehydrated, embedded, sectioned (5 μm), and stained with hematoxylin‐eosin and fast green‐safranin‐O staining solution. The sections were allowed to dry completely, and then sealed with neutral gum, and image acquisition was carried out using the Pannoramic 250 digital section scanner (3DHISTECH, Hungary). Histopathological assessment of the tendon‐bone junction was conducted using the semiquantitative Bonar scale. This scale encompasses four subscales, graded on a scale of 0 to 3, where 0 signifies normal, and 3 indicates maximal abnormality: 1 pertains to tenocyte morphology and proliferation, 2 assesses the presence or absence of ground substance, 3 evaluates collagen bundle characteristics, and 4 examines vascularity. The Bonar grade was determined as the cumulative sum of the subscale scores. The Image J software conducts semi‐quantitative analysis of the positive expression area in the safranin/green staining images.

### 2.6 Enzyme‐Linked Immunosorbent Assay (ELISA)

The concentration of Osteocalin (OCN), calcium (Ca), alkaline phosphatase (ALP), and Acetyl‐CoA were detected using ELISA kits, with corresponding kits.

### 2.7 Flow Cytometry

Mouse tendon bone junction tissues were rinsed with ice‐cooled PBS and homogenized, and centrifuged to obtain cell precipitates. PBS was used to resuspend the cells, and F4/80 and CD86 were added to each tube, which were incubated at 4°C, protected from light, centrifuged at 1000 rpm for 5 min, and the supernatant was discarded. 200 μL of Fixation Buffer was added to each tube, and the tubes were incubated at room temperature for 20 min, and the tubes were centrifuged at 1000 rpm for 5 min, and the supernatant was discarded. 250 μL Intracellular Straining Perm Wash Buffer was added to each tube, 100 μL Intracellular Straining Perm Wash Buffer was added to resuspend the cells, CD206 was added to each tube, incubated at 4°C for 30 min, centrifuged at 1000 rpm for 5 min, the supernatant was discarded, 300 μL PBS was used to resuspend the cells, and the cells were analyzed by Cytoflex flow analyzer.

### 2.8 Immunofluorescence (IF) Evaluation

The dewaxed sections were blocked with 3% hydrogen peroxide to block endogenous peroxidase, followed by serum blocking, incubation with primary antibody [Sox9 (1:100), Collagen II (1:100)] overnight at 4°C, and secondary antibody (FITC‐labeled goat anti‐rabbit, 1:100) for 30 min at room temperature, followed by DAPI staining. Images were captured using a VS200 Olympus scanner (Japan) and analyzed by Image‐J Data Image Analysis System. In addition, the expression of F4/80, CD86, and CD206 in cell smears was assayed in the same way.

### 2.9 Real‐Time Reverse Transcription‐Quantitative Polymerase Chain Reaction (RT‐qPCR)

Briefly, tissues were homogenized and pulverized in a high‐speed low‐temperature mode, and total RNA was isolated; similarly, cellular samples were extracted for RNA. cDNA was synthesized using a reverse transcription kit. The mRNA expression levels for each gene were quantified using real‐time PCR. Primers were designed and synthesized by Shanghai Sangon Biotech. The expression levels of IL‐1β, IL‐6, Arg1, IL‐10, TGF‐β1, TGF‐β3, Cpt1a, and ACS were assessed, with β‐actin as the internal control. Primer sequences were listed in Table 1.

**Table 1:** Primer sequences used for RT‐qPCR.

Gene name	Forward (5′ to 3′)	Reverse (5′ to 3′)
β‐actin	CTACCTCATGAAGATCCTGACC	CACAGCTTCTCTTTGATGTCAC
IL‐1β	TCGCAGCAGCACATCAACAAGAG	AGGTCCACGGGAAAGACACAGG
IL‐6	CTCCCAACAGACCTGTCTATAC	CCATTGCACAACTCTTTTCTCA
Arg1	CATATCTGCCAAAGACATCGTG	GACATCAAAGCTCAGGTGAATC
IL‐10	TTCTTTCAAACAAAGGACCAGC	GCAACCCAAGTAACCCTTAAAG
TGF‐β1	GGACCGCAACAACGCCATCTATGAGA	CTTGGTTCAGCCACTGCCGTACAACT
TGF‐β3	ATCAAGAAGAAGAGGGTGGAAG	GTAAAGTGCCAGGACCTGATAG
Cpt1a	CTACATCACCCCAACCCATATT	GATCCCAGAAGACGAATAGGTT
ACS	AATATCTACCTGCGGAGTGAAG	TTCTTCGAAGGACCCTTGTAAG

### 2.10 Western Blotting Analysis

Total protein was obtained from cells using RIPA lysis buffer, and quantified with the BCA kit (Beyotime, China). It was then separated by 10% SDS‐PAGE gel electrophoresis and transferred onto a PVDF membrane (Sigma‐Aldrich, USA). This was followed by blocking in 5% skim milk for 1 h at room temperature and incubation with the following primary antibodies: PPAR‐γ (1:1000) and RXRA (1:2000). The membranes were washed and then incubated with biotinylated Goat Anti‐Rabbit IgG (H + L) HRP secondary antibody (1:5000, Affbiotech, China) for 1 h at room temperature. Finally, the protein bands were detected using an ECL assay kit and analyzed with Image J. The relative protein expression level was calculated with reference to the expression of GAPDH, the house keeping gene.

### 2.11 Statistical Analysis

The obtained data were analyzed using the Statistical Product and Service Solutions (SPSS) software (version 20.0) and presented as the mean ± standard deviation (SD). To determine whether the differences between the examined groups are significant, one‐way analyses of variance (ANOVAs) and Tukey post hoc tests were carried out. The Shapiro‐Wilk test of normality and the Levene's test for homogeneity of variances were used to verify the assumptions of normal distribution and homogeneity of variances. Differences were considered to be statistically significant for *p* < 0.05.

## 3 Results

### 3.1 Effect of DID on Interfacial Bone Formation and Structure in Tendon Bone Healing Mice

In the experimental stage, one mouse in the model group died. Therefore, the deceased mouse was excluded from subsequent analyses, resulting in a final sample size of *n *= 5 for the model group. Micro CT scanning revealed that the volume of neoplastic bone in the Sham group was similar to that of the H‐DID group and the DSF bone tunnel (Figure 1A), and the analysis of microstructural parameter data also suggested that the levels of BV/TV, BS, Tb.N, and BMD were significantly increased, and the level of Tb.Sp was significantly reduced after H‐DID treatment compared with that of the model group (Figure 1B), suggesting that DID effectively promoted new bone formation in tendon bone healing. Figure 1C demonstrates that the collagen fibers were disarranged, the nuclei of fibroblasts were consolidated, and the necrotic area and the surrounding synovial area were accompanied by varying degrees of inflammatory cell infiltration, fibroblastic tissue proliferation, and neocapillaris generation in the tendon bone junction tissues of the model group. This is consistent with the pathological score results shown in Figure 1E. However, these changes were attenuated in the H‐DID and DSF groups. Figure 1D shows that the OCN, ALP, and Ca of mice treated with DID and DSF were significantly higher compared with those of the model group.

**Figure 1:**
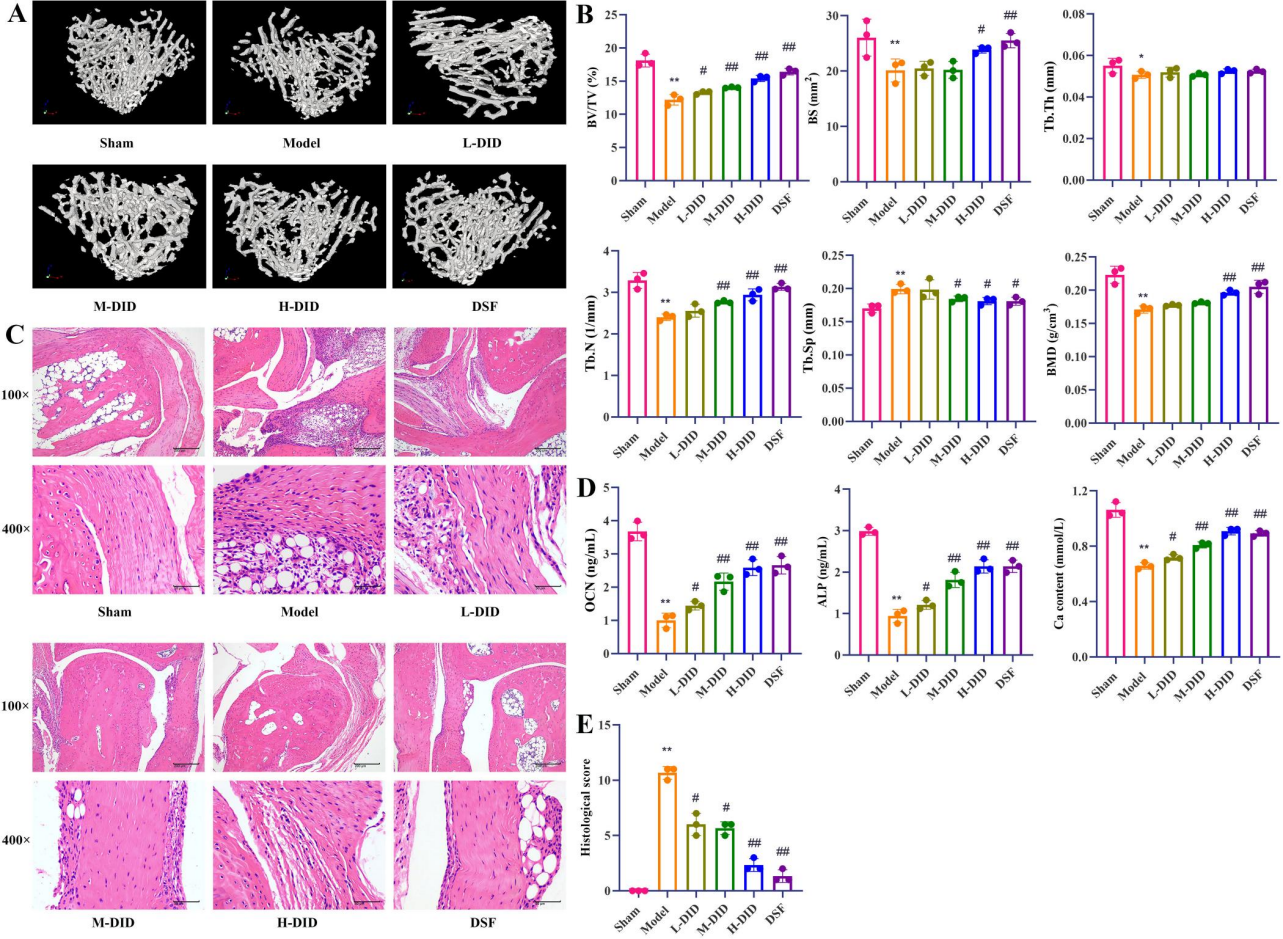
Effect of DID on interfacial bone formation and structure in tendon bone healing mice. (A) Micro CT view representative image; (B) levels of BV/TV, BS, Tb.Th, Tb.N, Tb.Sp, BMD; (C) representative image of HE staining of tendon‐bone junction tissue (100× or 400×, 200 µm, or 50 µm); (D) levels of OCN, ALP, and Ca in serum. (E) Quantification of the Bonar scale score based on the HE images. Data are expressed as the mean ± SD (*n* = 3 biological replicates). *p < 0.05, **p < 0.01 vs. sham group; #*p* < 0.05, ##*p* < 0.01 vs. model group.

### 3.2 Effect of DID on Fibrocartilage Formation at the Interface of Tendon Bone Healing Mice

Figure 2A,C shows that there was localized absence of cartilage layer and chondrocyte hyperplasia in tendon‐bone junction tissues of the model group, however, DID‐H and DSF significantly improved the above symptoms. The percentage of fibrous tissue area in the stained tissues by phloxine G staining showed consistent results. Immunofluorescence assay was used to measure the expression of cartilage markers Sox9 and Collagen II at the tendon‐bone interface, compared with the Sham group, the expression of Sox9 and Collagen II was reduced in the model group, while DID and DSF significantly promoted the expression of Sox9 and Collagen II (*p *< 0.01, Figure 2B,D). Furthermore, Figure 2E reveals that, compared with the Sham group, TGF‐β1 and TGF‐β3 were expressed at low levels at the tendon‐bone interface in the model group, and the expression of TGF‐β1 and TGF‐β3 was increased after DID and DSF treatment.

**Figure 2:**
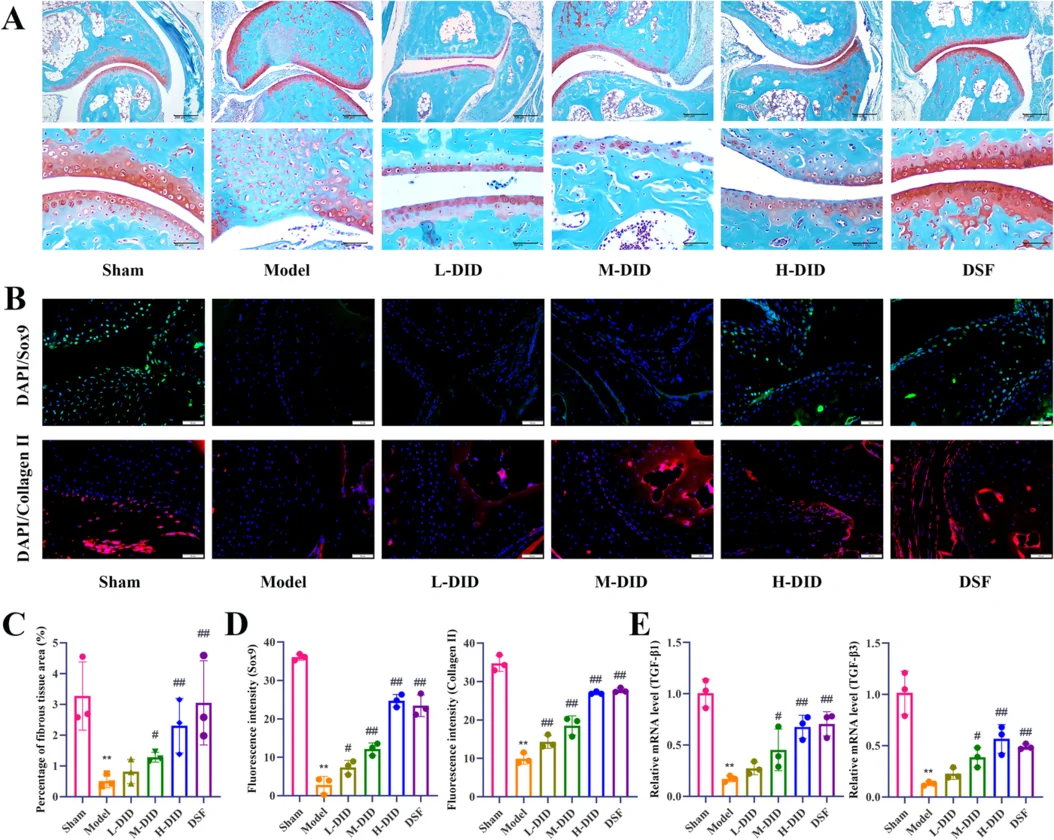
Effect of DID on fibrocartilage formation at the interface of tendon bone healing mice. (A) Safranin‐O/fast green staining of tendon‐bone junction tissue (100× or 400×, 200 µm, or 50 µm); (B) immunofluorescence assay was used to measure the expression of cartilage markers Sox9 and Collagen II at the tendon‐bone interface (20×, 50 µm); (C) percentage of fibrous tissue area within the Safranin‐O/fast green staining. (D) Expression of Sox9 and Collagen II at the tendon‐bone interface; (E) expression of TGF‐β1 and TGF‐β3 at the tendon‐bone interface was detected by qRT‐PCR. Data are expressed as the mean ± SD (*n* = 3 biological replicates). **p < 0.01 vs. sham group; #*p* < 0.05, ##*p* < 0.01 vs. model group.

### 3.3 Effect of DID on Macrophage Polarization in Tendon‐Bone Healing Mice

The impact of DID on macrophage polarization in tendon‐bone healing mice was investigated by analyzing the levels of macrophage‐associated factors. It was found that the levels of F4/80^+^CD86^+^, IL‐1β, and IL‐6 were elevated in the model mice, and the administration of low, medium, and high doses of DID decreased the levels of these factors in the model mice. Moreover, administration of medium and high doses of DID elevated the levels of F4/80^+^CD206^+^, Arg1, and IL‐10 in the model mice (*p *< 0.05, Figure 3), suggesting that the application of DID promotes the polarization of macrophages at the tendon‐bone interface towards M2 and reduces their polarization towards M1.

**Figure 3:**
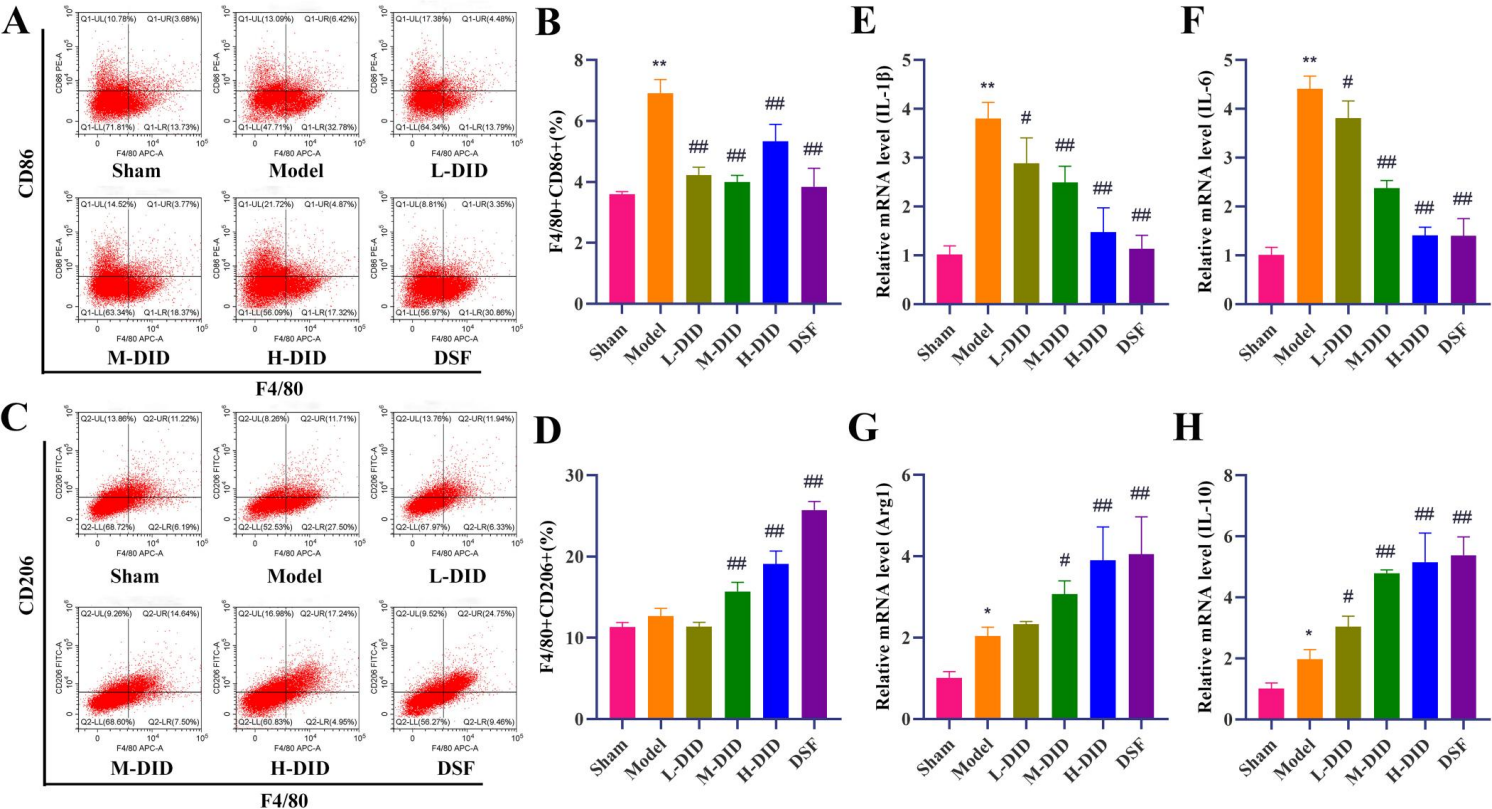
Effect of DID on macrophage polarization in tendon‐bone healing mice. (A) Representative images from flow cytometric analysis of F4/80^+^CD86^+^; (B) quantitative analysis of F4/80^+^CD86^+^; (C) representative images from flow cytometric analysis of F4/80^+^CD206^+^; (D) quantitative analysis of F4/80^+^CD206^+^; (E) levels of IL‐1β mRNA as measured by qRT‐PCR; (F) levels of IL‐6 mRNA as measured by qRT‐PCR; (G) levels of Arg1 mRNA as measured by qRT‐PCR; (H) levels of IL‐10 mRNA as measured by qRT‐PCR. Data are expressed as the mean ± SD (*n* = 3 biological replicates). *p < 0.05, **p < 0.01 vs. sham group; #*p* < 0.05, ##*p* < 0.01 vs. model group.

### 3.4 Effect of DID on Macrophage Polarization and PPAR Signaling‐Mediated FAO Pathway in BMDMs

To evaluate the impact of DID on macrophage polarization, we measured F4/80^+^CD86^+^ and F4/80^+^ CD206^+^ levels. The levels of F4/80^+^CD86^+^ were increased in BMDMs stimulated by LPS/IFN‐γ combination (*p *< 0.01, Figure 4A,B), and the levels of F4/80^+^CD206^+^ were increased in BMDMs induced by the IL‐4/IL‐13 combination (*p *< 0.01, Figure 4C,D), and DID treatment significantly reduced the levels of F4/80^+^CD86^+^ (*p* < 0.01). In addition, the levels of the PPAR signaling‐mediated fatty acid oxidation pathway were analyzed. The levels of CoA, Cpt1a, ACS, PPAR‐γ, and RXRA were significantly suppressed in the BMDMs (M1) group compared to the BMDMs (M0) group, whereas DID treatment promoted the levels of the above‐mentioned factors (*p *< 0.05, Figure 4E–J), demonstrating that DID induced macrophage M2 polarization and promoted the fatty acid oxidation pathway mediated by PPAR signaling.

**Figure 4:**
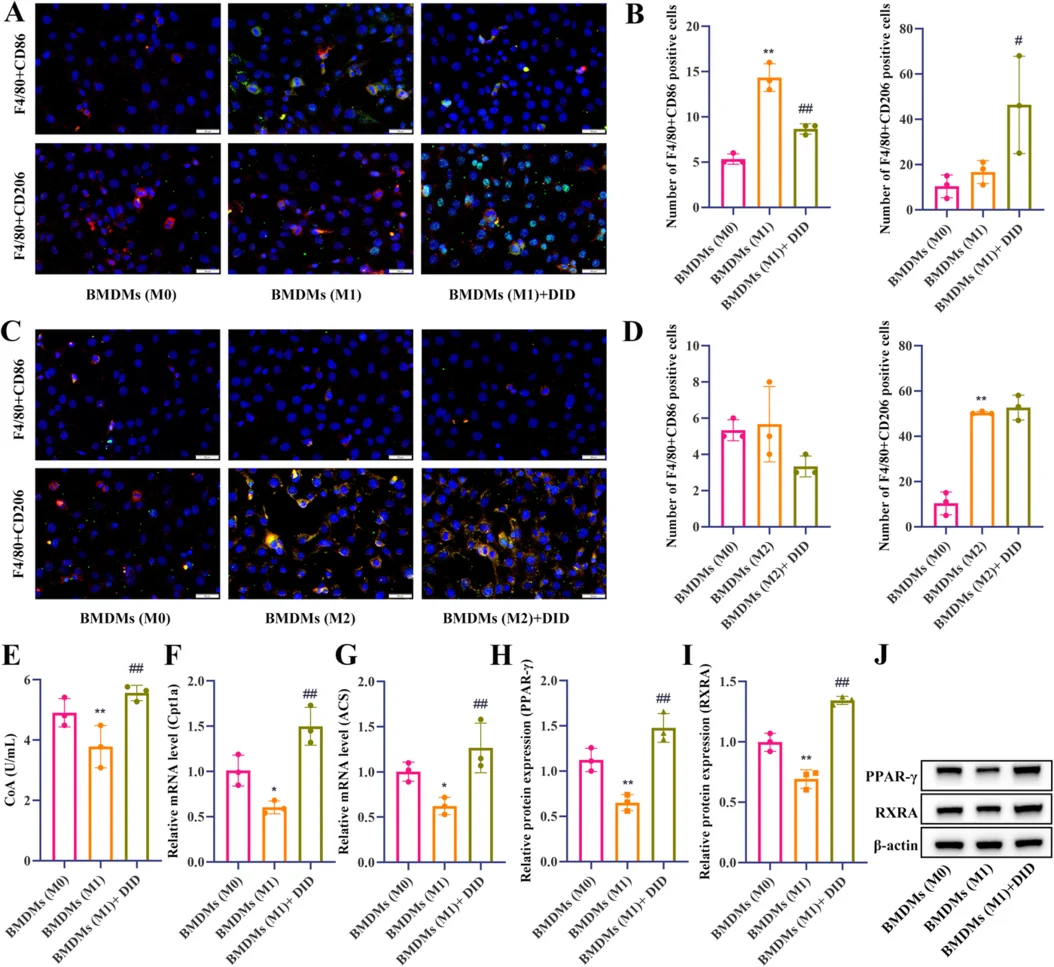
Effect of DID on macrophage polarization and PPAR signaling‐mediated FAO pathway in BMDMs. (A) Representative pictures of immunofluorescence staining (20×, 50 μm) of F4/80^+^CD86^+^ and F4/80^+^CD206^+^ in response to LPS/IFN‐γ co‐stimulation; (B) quantitative analysis of F4/80^+^CD86^+^ and F4/80^+^CD206^+^; (C) representative pictures of immunofluorescence staining (20×, 50 μm) of F4/80^+^CD86^+^ and F4/80^+^CD206^+^ in response to IL‐4/IL‐13 co‐stimulation; (D) quantitative analysis of F4/80^+^CD86^+^ and F4/80^+^CD206^+^; (E) levels of CoA were detected by ELISA; (F) levels of Cpt1a mRNA as measured by qRT‐PCR; (G) levels of ACS mRNA as measured by qRT‐PCR; (H) levels of PPAR‐γ protein as measured by Western blotting; (I) levels of RXRA protein as measured by Western blotting; (J) protein bands were analyzed by Western blotting for PPAR‐γ and RXRA. Data are expressed as the mean ± SD (*n* = 3 biological replicates). *p < 0.05, **p < 0.01 vs. BMDMs (M0) group; #*p* < 0.05, ##*p* < 0.01 vs. BMDMs (M1) group.

### 3.5 Validation of PPAR Signaling‐Mediated FAO Pathway Involved in DID‐Induced Macrophage Polarization in BMDMs

To verify that the PPAR signaling‐mediated FAO pathway is involved in DID‐induced macrophage polarization in BMDMs, PPAR‐γ antagonists and FAO inhibitors were used in the experiments. As shown in Figure 5A, we first verified the inhibitory effect of the PPAR‐γ antagonist, and GW9662 significantly inhibited the promotion of PPAR‐γ and RXRA expression in BMDMs (M1) by DID (*p *< 0.01). Compared with the BMDMs (M1) + DID group, the BMDMs (M1) + DID + GW9662 and the BMDMs (M1) + DID+ Etomoxir groups significantly decreased the levels of CoA (Figure 5B), F4/80^+^CD206^+^ (Figure 5C), Arg1, and IL‐10 (Figure 5D, *p* < 0.05), and significantly increased the levels of F4/80^+^CD86^+^ (Figure 5C), IL‐1β, and IL‐6 (Figure 5D, *p* < 0.05), suggesting that DID may be involved in macrophage polarization in BMDMs by promoting the FAO pathway mediated by PPAR signaling.

**Figure 5:**
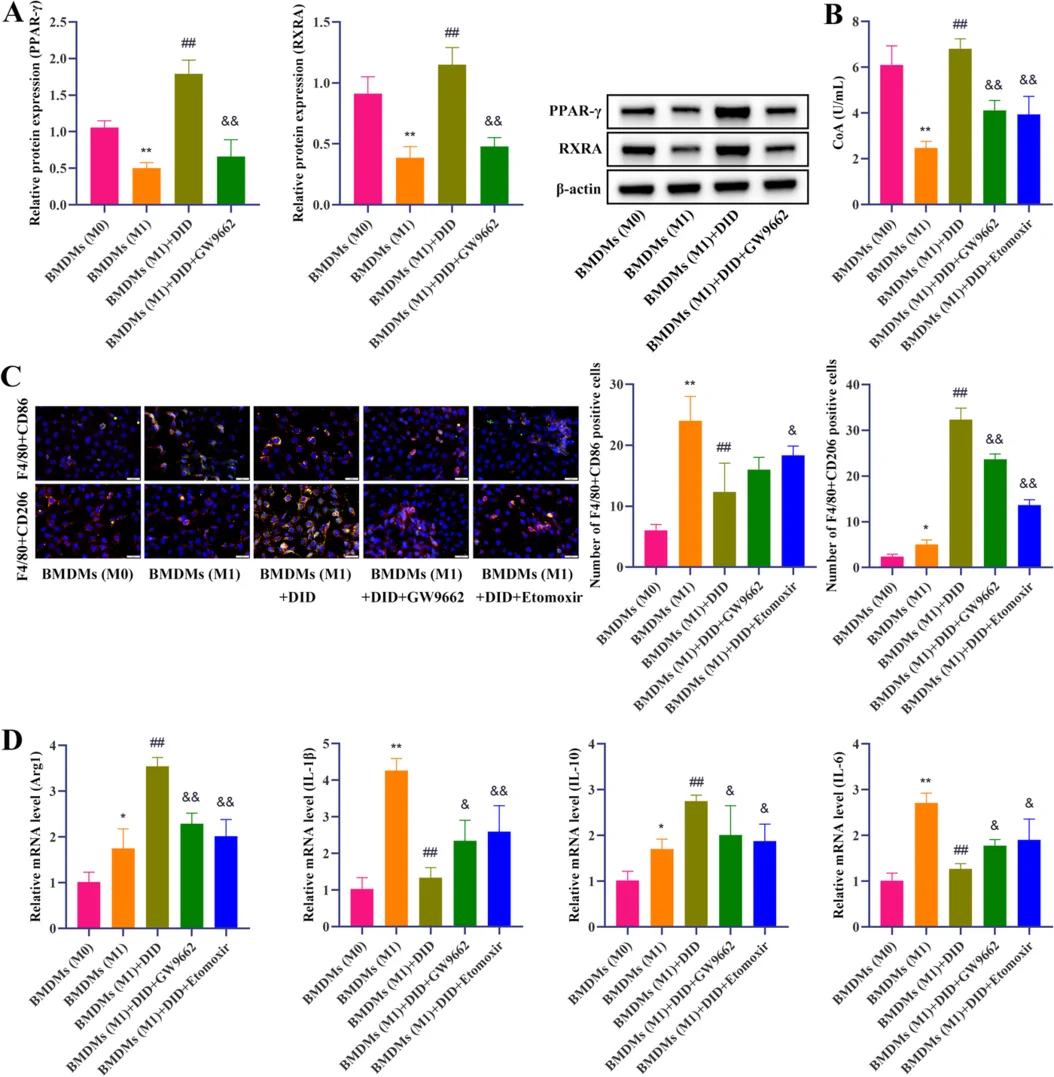
Validation of PPAR signaling‐mediated FAO pathway involved in DID‐induced macrophage polarization in BMDMs. (A) Levels of PPAR‐γ and RXRA protein as measured by Western blotting; (B) levels of CoA were detected by ELISA; (C) immunofluorescence staining (20×, 50 μm) of F4/80^+^CD86^+^ and F4/80^+^CD206^+^; (D) levels of Arg1, IL‐1β, IL‐10, and IL‐6 mRNA as measured by qRT‐PCR. Data are expressed as the mean ± SD (*n* = 3 biological replicates). *p < 0.05, **p < 0.01 vs. BMDMs (M0) group; #*p* < 0.05, ##*p* < 0.01 vs. BMDMs (M1) group; &*p* < 0.05, &&*p* < 0.01 vs. BMDMs (M1) + DID group.

## 4 Discussion

Tendon‐bone junction injuries are very common in clinical practice, and surgical reconstruction is the most effective treatment for the vast majority of patients. However, after reconstruction, the tendon‐bone junction has poor self‐repairing ability, forms a large amount of scar tissue at the tendon‐bone interface, and has low biomechanical strength, which leads to a high re‐break rate after surgery [4, 21]. The study of the mechanism of tendon‐bone junction injury healing is an urgent scientific problem in the field of tendon‐bone healing research [22]. Previous studies have confirmed that DID has detoxifying and decongestive, antibacterial, and anti‐inflammatory effects, and can regulate the balance between M1 and M2‐like macrophages [16, 18]. However, its role and mechanism of application to tendon‐bone healing remain unclear. Therefore, we explored the role of DID in tendon‐bone healing. In this study, DID promoted fibrocartilage formation in tendon‐bone junction tissues, drove macrophage polarization towards M2 type, and affected macrophage polarization by promoting PPAR signaling‐mediated FAO pathway.

There are many models of tendon‐bone junction healing that correspond to different injury sites in the clinic. Small animals, such as rats and mice, can be used for the reconstruction of the Achilles tendon junction, and the number of animals can easily meet the requirements of experiments, and there is a complete range of antibodies that can be used in mouse studies [23]. Therefore, mice were chosen as the experimental animals for this study, taking into account the needs of the later experiments. The study also showed that this model was similar to the pattern of change after rotator cuff reconstruction and could reflect the process of tendon‐bone healing through the morphological observation at different time points after the operation [24]. Therefore, the mouse Achilles tendon‐Achilles bone reconstruction was chosen as the animal model for tendon‐bone healing in this study. Although this model is applicable for studying the healing process of tendons and bones, it cannot fully reflect the biomechanics, vascular supply, and stress conditions of the attachment points of the rotator cuff. This may limit the direct application of its conclusions to rotator cuff repair. It is worth noting that all the mice in the postoperative group received a local injection of penicillin to prevent infection. This ensured consistency among the groups. However, penicillin, as a broad‐spectrum antibiotic [25], may have certain interactions with the anti‐inflammatory and potential antibacterial properties of DID. This potential synergistic or antagonistic effect may affect the interpretation of the experimental results, and it is necessary to further explore this in future studies.

Bone formation, especially new bone formation and remodeling around tendon attachment points, is a critical step in the tendon‐bone healing process [26]. Our results found that DID effectively promoted new bone formation in tendon‐bone healing. Han et al. [27] found that Total flavonoids of Rhizoma drynariae (TFRD) enhanced the viability and osteogenic differentiation of BMSCs in vitro and increased ALP activity and OCN secretion. Icariin (ICA) significantly increased serum ALP and OCN protein expression levels, and bone mass was significantly enhanced [28]. In the present study, DID significantly increased serum levels of OCN, ALP, and Ca, which is similar to the results reported above. In addition, HE and safranin‐O/fast green staining of tendon‐bone junction tissues suggested that DID promoted fibrocartilage formation. This suggests that DID has a promoting effect on tendon‐bone healing.

Inflammatory responses are widely involved in the regeneration and repair processes of various tissues and organs in the body, and studies have shown that macrophages are essential inflammatory cells that are critical in the process of tissue healing [5]. M2‐type macrophages have been demonstrated in multiple tissue studies to be key cells in promoting tissue repair, and they secrete anti‐inflammatory factors such as Arg1, IL‐10, and TGF‐β to promote tissue repair [29, 30]. In contrast, M1‐type macrophages secrete pro‐inflammatory factors such as IL‐1β, TNF‐α, and IL‐6, which play a negative role in cartilage regeneration and repair [31]. Studies have shown that local administration of IL‐4 and IL‐13 at the fracture site promotes macrophage polarization towards the M2 type, leading to an improvement in the local inflammatory microenvironment and thus promoting fracture healing [32]. In addition, it was shown that M2‐type macrophages promoted cartilage repair by secreting TGF‐β1 and TGF‐β3 [33]. In the present study, we found that DID effectively induced an increase in M2‐type macrophages (CD206^+^) and an increase in the expression of anti‐inflammatory factors (Arg1, IL‐10) in tissues of the tendon bone junction, whereas the expression of M1‐type macrophages (CD86^+^) and pro‐inflammatory factors (IL‐1β, IL‐6) was significantly reduced, suggesting that DID induces macrophage polarization to M2 type at the tendon‐bone healing site.

To further clarify the regulatory effect of DID on macrophage polarization, we obtained mature BMDMs and induced them into M1‐ and M2‐type polarized macrophages in vitro, and examined the role of DID in this process. The results showed that DID had no direct effect on M2‐type macrophage polarization, whereas the in vivo findings suggested that DID was able to simultaneously down‐regulate M1‐type macrophages and up‐regulate M2‐type macrophages, an intriguing phenomenon that suggests whether DID promotes some kind of transformational process in macrophages. Studies have found that macrophages, once activated, maintain plasticity and can transform from one phenotype to another in response to environmental signals [34]. We demonstrated that DID significantly down‐regulated M1 (CD86^+^) and up‐regulated M2‐type macrophages (CD206^+^), and associated factor mRNA expression under M1 polarization conditions, proving that DID promotes the conversion from M1 to M2. In addition, PPAR‐γ directly promotes the differentiation of bone marrow MSCs to osteoblasts, which indirectly leads to a decrease in osteogenesis, and the activation of PPAR‐γ and its regulatory genes contributes to an increase in FAO, on which M2‐type macrophages depend for energy [13, 35]. In the present study, DID increased the levels of PPAR‐γ, RXRA, and CoA, and the up‐regulation of M2‐type macrophages by DID was inhibited after the addition of PPAR‐γ antagonists and FAO inhibitors. These results suggest that DID may drive macrophage polarization toward M2 by promoting the PPAR‐mediated FAO pathway, which in turn promotes tendon‐bone healing.

## 5 Conclusions

In summary, this study reveals that DID promotes fibrocartilage formation in tendon‐bone junction tissues, drives macrophage polarization towards M2 type, and may modulate macrophage polarization by promoting PPAR signaling‐mediated FAO pathway. Our results lay the foundation for the application of DID.

### 5.1 Study Limitation

However, there are several limitations to our study. For example, biomechanical tests were not conducted, and the mechanism of DID was not validated using PPAR‐γ antagonists/FAO inhibitor in vivo experiments. Additionally, all experimental animals received uniform local penicillin injections postoperatively to prevent infection. Although this measure ensured homogeneity for intergroup comparisons, future studies should establish an “antibiotic‐free” group to completely exclude its potential influence. In the future, we will conduct more in‐depth animal studies to investigate the effects and mechanisms of DID on tendon‐bone healing. In addition, this study only used male mice. Sex may influence the inflammatory response, bone metabolism, and tendon healing. Future studies will need to use female animals for verification.

## Author Contributions

**Xiaojun Ma:** conceptualization, writing – original draft, data curation. **Jia Shen:** data curation. **Jun Ma:** writing – review and editing. **Hangyu Li:** data curation. **Yang Li:** data curation. **Jun Wan:** conceptualization, writing – review and editing, funding acquisition.

## Conflicts of Interest

The authors declare no conflicts of interest.

## Supporting information


Supporting File 1

Supporting File 2

## Data Availability

The data sets used and analyzed during the current study are available from the corresponding author on reasonable request.
