# Supplementary material for: Didymin may Enhance Tendon‐Bone Healing Partly by Promoting PPAR‐Mediated Fatty Acid Oxidation and Macrophage M2 Polarization
Source: Immun Inflamm Dis. 2026 Sep 29;14(9):e70485. doi: 10.1002/iid3.70485 (PMC13621933; doi:10.1002/iid3.70485)
Supplement: Supplementary file 2 [file IID3-14-e70485-s002.pdf]

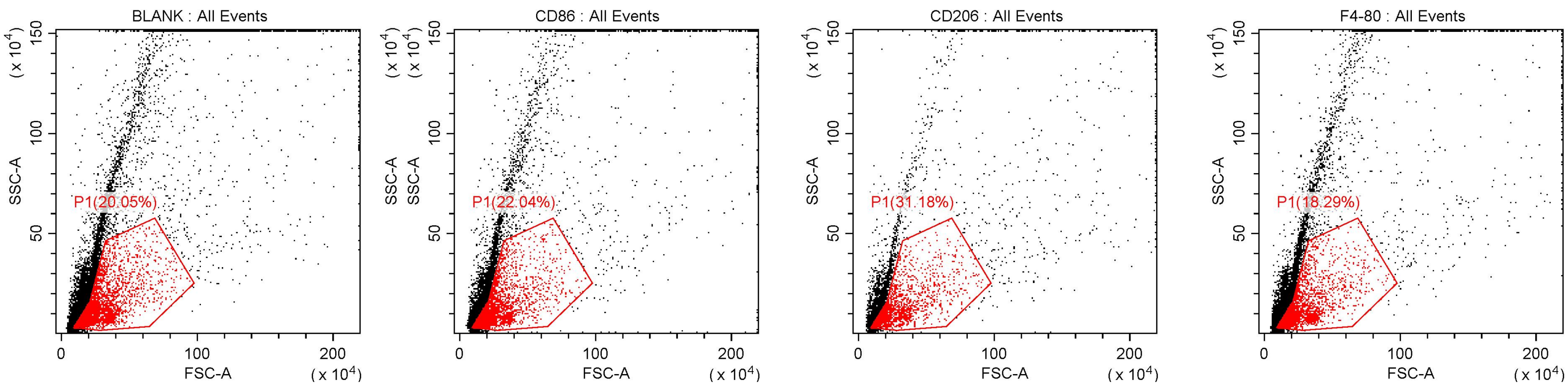
BLANK : All Events
CD86 : All Events
CD206 : All Events
F4-80 : All Events
P1(20.05%)
P1(22.04%)
P1(31.18%)
P1(18.29%)
SSC-A
FSC-A
(x 10^4)
0
50
100
150
200

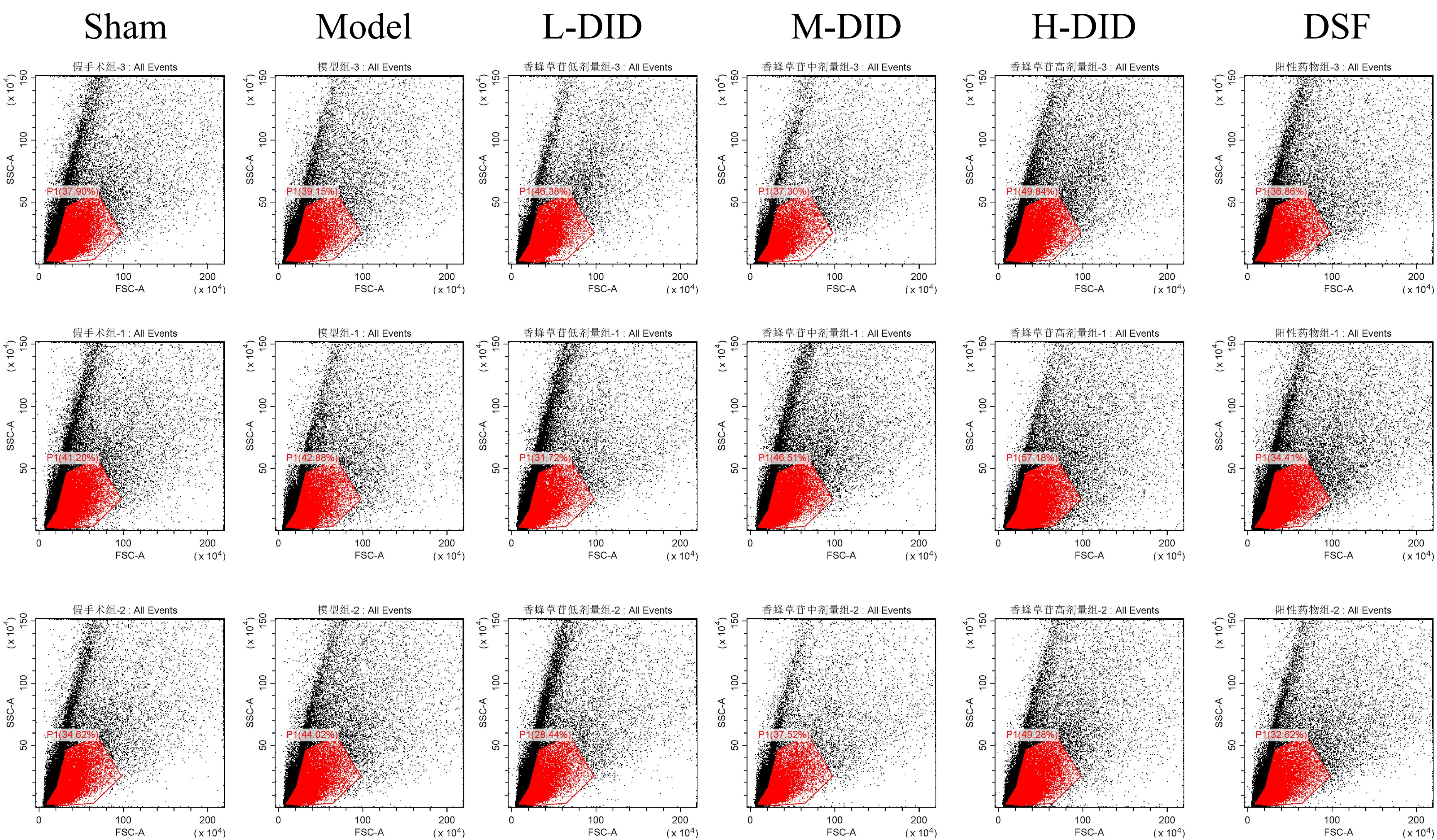
Sham
Model
L-DID
M-DID
H-DID
DSF
假手术组-3 : All Events
P1(37.90%)
模型组-3 : All Events
P1(39.15%)
香蜂草苷低剂量组-3 : All Events
P1(46.38%)
香蜂草苷中剂量组-3 : All Events
P1(37.30%)
香蜂草苷高剂量组-3 : All Events
P1(49.84%)
阳性药物组-3 : All Events
P1(36.86%)
假手术组-1 : All Events
P1(41.20%)
模型组-1 : All Events
P1(42.88%)
香蜂草苷低剂量组-1 : All Events
P1(31.72%)
香蜂草苷中剂量组-1 : All Events
P1(46.51%)
香蜂草苷高剂量组-1 : All Events
P1(57.18%)
阳性药物组-1 : All Events
P1(34.41%)
假手术组-2 : All Events
P1(34.62%)
模型组-2 : All Events
P1(44.02%)
香蜂草苷低剂量组-2 : All Events
P1(28.44%)
香蜂草苷中剂量组-2 : All Events
P1(37.52%)
香蜂草苷高剂量组-2 : All Events
P1(49.28%)
阳性药物组-2 : All Events
P1(32.62%)
SSC-A
(x 10^4)
FSC-A
(x 10^4)
0
50
100
150
200
